# Cultivar differences in carbon and nitrogen accumulation, balance, and grain yield in maize

**DOI:** 10.3389/fpls.2022.992041

**Published:** 2022-09-09

**Authors:** Qiang Li, Yun Ren, Hao Fu, Zhexin Li, Fanlei Kong, Jichao Yuan

**Affiliations:** ^1^Chongqing Key Laboratory of Economic Plant Biotechnology, Collaborative Innovation Center of Special Plant Industry in Chongqing, Institute of Special Plants, Chongqing University of Arts and Sciences, Chongqing, China; ^2^Key Laboratory of Crop Ecophysiology and Farming System in Southwest China, Ministry of Agriculture, Sichuan Agricultural University, Ya’an, Sichuan, China

**Keywords:** maize, nitrogen accumulation, carbon accumulation, C/N ratio, grain yield

## Abstract

The balance of carbon (C) and nitrogen (N) metabolism influences plant growth and development as well as yield. A two-year field experiment was conducted in a hilly region in southwest China in 2019–2020 to investigate the correlation between the accumulation and balance of C and N, as well as the grain yield of maize cultivars with contrasting N efficiencies. Using Zhenghong 311 (ZH 311) and Xianyu 508 (XY 508) as research sources, the differences in C and N accumulation and balance in maize cultivars with contrasting N efficiencies were compared to analyze the correlation between the accumulation and balance of C and N with grain yield. According to the results, the ZH 311 cultivar had higher C and N accumulation in each stage and grain yield than the XY 508 cultivar, while the C/N ratio in each stage and organ was significantly lower in ZH 311 than in XY 508, with the greatest difference occurring in the silking stage and leaf, indicating that the N-efficient cultivar ZH 311 had evident advantages in accumulation and balance of C and N and grain yield than the N-inefficient cultivar XY 508. Moreover, the C and N accumulation and grain yield increased significantly with N application, while the C/N ratio in each stage and organ decreased significantly with N application, but the differences between ZH 311 and XY 508 increased first and then decreased with the increase of N level, the optimum N level when obtaining the highest grain yield of ZH 311 (273.21 kg ha^–1^) was significantly lower than that of XY 508 (355.88 kg ha^–1^). Furthermore, grain yield was positively correlated with C (*R*^2^ = 0.9251) and N (*R*^2^ = 0.9033) accumulation, affected by pre-anthesis N (*R*^2^ = 0.9198) and post-anthesis C (*R*^2^ = 0.8632) accumulation, and negatively correlated with the C/N ratio (*R*^2^ = 0.7664), with the highest correlation between grain yield and the C/N ratio in silking stage (*R*^2^ = 0.7984) and leaf (*R*^2^ = 0.7616). In conclusion, the N-efficient cultivar ZH 311 could better coordinate the C and N balance of the plant, especially the C and N balance in the silking stage and leaf, promote photosynthetic product storage and transport, prolong the leaf function period, and make the pre-anthesis and post-anthesis C and N accumulation of ZH 311 significantly higher than those of XY 508, allowing higher grain yields.

## Introduction

Maize (*Zea mays* L.) is the world’s largest food crop (Jiao et al., 2017). Total maize production has exceeded 1 billion tons, accounting for 41% of global food production. Maize production is crucial to global food security (Hake and Ross-Ibarra, 2015; Wang et al., 2016). Nitrogen (N) is a key limiting factor for crop growth and yield as it is not only a component of proteins, nucleic acids and chlorophyll in plants, but it is also involved in metabolic processes such as photosynthesis and respiration (Ge et al., 2020; Girón-Calva et al., 2021). Increasing N fertilizer application can effectively increase maize yield, so N fertilizer is often excessively applied during maize production (Correndo et al., 2021; Nafziger et al., 2021). Excessive application of N fertilizer will not only decrease maize yield and quality, but also increase production costs and waste resources, as well as cause a series of environmental problems such as soil acidification, and soil and water pollution (Chen et al., 2021). Under the premise of reducing N fertilizer application, increasing maize yield per unit area is critical to ensuring food security and alleviating environmental problems (Ladha et al., 2016). The selection of N-efficient maize cultivars is an important way to increase the yield per unit area and solve the abuse of N fertilizer (Chun et al., 2005).

Carbon (C) and N metabolism are the two most important metabolic processes in plants, which occur throughout plant growth and development (Commichau et al., 2006; Hu et al., 2017). The coordination of C and N metabolism not only influences plant growth and development, but also determines its yield (Butterly et al., 2015; Dong et al., 2017). The concentration of C and N and the C/N ratio in plants greatly affect the metabolic process of crops, which then affect the accumulation of C and N in plants and, ultimately, the crop yield (Zhang et al., 2016; Xie et al., 2018). Chang et al. (2017) demonstrated that maize cultivars with well-coordinated C and N metabolism can balance the contradiction between grain filling and vegetative organ senescence in the C and N transport process, resulting in a high grain yield. Maize concentration of C and N in vegetative organs, as well as the C/N ratio, were strongly related to grain yield and kernels per ear (Shen et al., 2007). Increasing the transport of pre-anthesis C and N accumulation of maize could ensure yield under N deficiency, and that increasing N fertilizer application can improve the contribution rate of post-anthesis C and N accumulation to grain to obtain a high yield (Wu et al., 2021). Previous studies on the effect of C and N metabolism on maize yield has mainly focused on changes in enzyme activity of C and N metabolism and the concentration of C and N (Chang et al., 2017; Xie et al., 2018; Li et al., 2021), while the cultivar differences in carbon and nitrogen accumulation, balance, and grain yield in maize remains unknown.

Therefore, to clarify the differences in carbon and nitrogen accumulation, balance, and grain yield in maize, this study used N-efficient cultivar Zhenghong 311 (ZH 311) and N-inefficient cultivar Xianyu 508 (XY 508) as experimental materials to study the differences in C and N accumulation and balance in maize cultivars with contrasting N efficiencies (Li et al., 2017). Furthermore, identifying the correlation between accumulation and balance of C and N and grain yield to provide a theoretical basis and technical support for the breeding and cultivation management of N-efficient maize cultivars.

## Materials and methods

### Experimental materials

The N-efficient maize cultivar ZH 311 and the N-inefficient maize cultivar XY 508 were used as experimental materials. Both cultivars are common in southwest China and have similar growth periods of approximately 120 days.

### Experimental design

**FIGURE 1:**
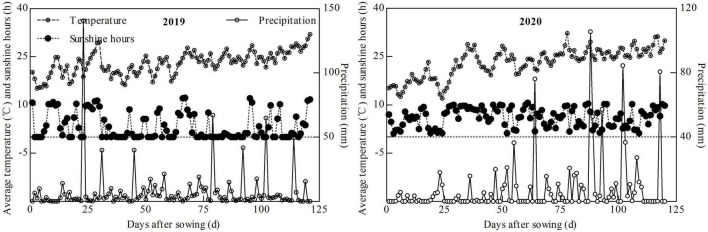
Meteorological factors during maize growth.

The experiment was conducted in the hilly area of Yongchuan (29°21′N, 105°54′E), Chongqing, China, between 2019 and 2020. This region has a warm and humid subtropical monsoon climate, as demonstrated by the meteorological factors that affect maize growth in Figure 1. The basic soil was sampled from the 0–30 cm soil layer. This was a typical purple soil and the physicochemical property show in Table 1.

**TABLE 1:** Basic physicochemical property of the tested soil.

Years	Organic matter (g⋅kg^–1^)	Total N (g⋅kg^–1^)	Total P (g⋅kg^–1^)	Total K (g⋅kg^–1^)	Alkali hydrolysable N (mg⋅kg^–1^)	Olsen-P (mg⋅kg^–1^)	Exchangeable K (mg⋅kg^–1^)	pH
2019	16.14	1.63	0.62	11.55	48.72	2.68	145.21	7.92
2020	13.97	1.55	0.49	9.74	39.77	2.37	131.19	7.63

The experiment consisted of 24 randomized block plots with three replications. The two factors were maize cultivars (ZH 311 and XY 508) and N levels, with four treatments: N 0: 0 kg ha^–1^, N 120: 120 kg ha^–1^, N 240: 240 kg ha^–1^, N 360: 360 kg ha^–1^. Plots were in 5 m × 8 m size (40 m^2^ total). At the sowing and pre-silking stages, N fertilizer with urea was applied in two equal split plots. In all treatments, 600 kg ha^–1^ of superphosphate and 150 kg ha^–1^ of potassium chloride were applied as base fertilizers. Cultivars were planted in 1.4 m and 0.6 m wide and narrow rows, respectively, with 52,500 plants ha^–1^. Other management measures were similar to those used in local maize production.

### Sampling and measurements

At the jointing stage (JS, 43 and 42 days after sowing in 2019 and 2020), silking stage (SS, 77 and 76 days after sowing in 2019 and 2020), and maturity stage (MS, 122 and 121 days after sowing in 2019 and 2020), five plants with an average height were sampled from the middle of each plot, and samples were divided into root, stems + sheath, leaf lamina, and ear. All samples were desiccated for 30 minutes at 105 °C, then at 80 °C until a constant weight was reached, then weighed, powdered, and passed through a 60-mesh sieve. The contents of total C and total N were determined using an element analyzer (Vario Max CN, elemental, Germany). The grain yield and 1,000-kernel weight were determined using all but the border rows in a 10-m^2^ area within each plot. The moisture content of the grain yield and kernel weight was adjusted to 14.0%. Yield components (row numbers, kernels per row, and kernels per ear) were determined from 20 sequential plants per plot.

### Data analysis

The data was analyzed using the least significant difference method using the SPSS software package, Version 20.0 (IBM Corp., New York, United States) to test whether significant differences existed between the N levels and cultivars. The significance level was set at *p* < 0.05, and GraphPad Prism 5.0 software was used for drawing. SPSS also used for correlation analysis.

## Results

### Differences in maize nitrogen and carbon accumulation

Cultivar, N level, and year all had significant (*p* < 0.01) effects on maize N accumulation and N harvest index (NHI), and there was a significant (*p* < 0.01) interaction effect between cultivar and N level on N accumulation and NHI (Table 2). The N accumulation in each stage of ZH 311 was significantly higher than that of XY 508 in both years, while the NHI of ZH 311 was significantly lower than that of XY 508. In 2019 and 2020, the N accumulation in the jointing stage of ZH 311 was higher than that of XY 508 by 15.37 and 5.99%, respectively. The silking stage was higher by 21.24 and 22.53%, the maturity stage was higher by 30.99 and 24.55%, and the NHI was lower by 5.67 and 6.67%, respectively. The N accumulation in each stage of maize increased significantly with N fertilization application, whereas the NHI decreased, but there were evident differences between cultivars. The N accumulation in each stage of ZH 311 increased first and then decreased with the increase of N application, which was highest in N 240 in both years, whereas the N accumulation in each stage (except for the jointing stage in 2020) of XY 508 increased with the increase of N application, which was the highest in N 360 in both years. Therefore, the difference in N accumulation between ZH 311 and XY 508 increased first and then decreased with the increase of N application, which was the greatest difference in N 240 in both years. The N accumulation in maturity stage of ZH 311 was higher than that of XY 508 by 38.13 and 31.31% in 2019 and 2020, respectively.

**TABLE 2:** Differences of N accumulation and NHI under different treatments.

Cultivars	Nitrogen rate	Jointing stage (kg ha^–1^)		Silking stage (kg ha^–1^)		Maturity stage (kg ha^–1^)		N harvest index	
		2019	2020	2019	2020	2019	2020	2019	2020
	N 0	18.58 f	23.11 g	66.22 f	55.01 f	104.17 d	100.51 d	0.50 d	0.58 bc
	N 120	31.38 d	34.86 e	101.19 d	87.46 d	162.73 b	136.58 c	0.51 cd	0.57 cd
ZH 311	N 240	38.90 a	44.28 a	147.54 a	117.82 a	205.02 a	180.39 a	0.50 d	0.55 de
	N 360	36.23 b	37.46 d	128.49 b	114.10 a	194.91 a	175.04 a	0.49 d	0.54 e
	Mean	**31.27 A**	**34.93 A**	**110.86 A**	**93.60 A**	**166.71 A**	**148.13 A**	**0.50 B**	**0.56** B
	N 0	15.17 g	19.34 h	52.77 g	41.50 g	80.20 e	74.32 e	0.56 a	0.62 a
	N 120	27.73 e	33.53 f	85.08 e	67.96 e	113.46 d	98.88 d	0.51 bcd	0.60 ab
XY 508	N 240	31.62 d	40.11 b	108.02 d	93.52 c	148.42 c	137.37 c	0.52 bc	0.58 bc
	N 360	33.92 c	38.83 c	119.90 c	102.55 b	167.00 b	165.16 b	0.53 b	0.60 ab
	Mean	**27.11 B**	**32.95 B**	**91.44 B**	**76.38 B**	**127.27 B**	**118.93 B**	**0.53 A**	**0.60 A**
*F* value	Years (Y)	282.36**		231.03**		80.15**		285.74**	
	Cultivar (C)	117.90**		296.67**		520.98**		85.01**	
	Nitrogen (N)	973.72**		793.65**		693.20**		9.74**	
	Y × C	15.01**		1.08^ns^		11.59**		0.43^ns^	
	Y × N	8.11**		4.44*		5.50**		1.69^ns^	
	C × N	15.02**		20.35**		23.84**		4.92**	
	Y × C × N	2.49^ns^		4.28*		2.11^ns^		1.82^ns^	

Values followed by different lowercase letters are significantly different with *p* < 0.05; within cultivars, values with different uppercase letters are significantly different with *p* < 0.05 according to the least significant difference test. **p < 0.01; *p < 0.05; ^ns^Not significant. The bold values are the mean value of one cultivar in different N treatments.

Cultivar, N level, and year all had significant (*p* < 0.01) effects on C accumulation in each stage and the C harvest index (CHI) of maize, and there was a significant (*p* < 0.01) interaction effect between cultivar and N level on C accumulation and CHI (Table 3). The C accumulation in each stage of ZH 311 was significantly higher than that of XY 508, while the CHI of ZH 311 was significantly lower than that of XY 508. In 2019 and 2020, the C accumulation in the jointing stage of ZH 311 was higher than that of XY 508 by 18.72 and 6.63%, respectively. The silking stage was higher by 13.84 and 16.14%, the maturity stage was higher by 29.51 and 22.34%, and the CHI was lower by 4.17 and 6.25%, respectively. The N application significantly increased the C accumulation in each stage of maize, but had no significant effect on the CHI. The C accumulation in each stage of ZH 311 increased first and then decreased with the increase of N application, which was highest in N 240 in both years, while the C accumulation in each stage (except for the jointing stage in 2019) of XY 508 increased with the increase of N application, which was highest in N 360 in both years. With increasing N application, the difference in C accumulation between ZH 311 and XY 508 increased at first, then decreased, and the difference was greatest in N 240 in both years. The C accumulation in the maturity stage of ZH 311 was higher than that of XY 508 by 34.19 and 29.11% in 2019 and 2020, respectively.

**TABLE 3:** Differences of C accumulation and CHI under different treatments.

Cultivars	Nitrogen rate	Jointing stage (kg ha^–1^)		Silking stage (kg ha^–1^)		Maturity stage (kg ha^–1^)		C harvest index	
		2019	2020	2019	2020	2019	2020	2019	2020
	N 0	309.94 de	390.75 e	1711.89 d	2072.86 e	4649.57 e	4279.12 d	0.46 abc	0.43 c
	N 120	335.00 c	433.32 c	2372.74 b	2499.92 c	6241.84 b	5186.75 b	0.47 abc	0.45 b
ZH 311	N 240	412.31 a	504.76 a	2764.71 a	2791.31 a	6980.10 a	5837.16 a	0.45 c	0.44 c
	N 360	385.39 b	414.70 d	2382.82 b	2652.24 b	6435.33 b	5568.12 a	0.46 bc	0.47 ab
	Mean	**360.66 A**	**435.88 A**	**2308.04 A**	**2504.08 A**	**6076.71 A**	**5217.79 A**	**0.46 B**	**0.45 B**
	N 0	267.22 f	319.72 f	1557.25 e	1788.95 f	3661.19 f	3314.15 e	0.48 a	0.48 a
	N 120	295.17 e	411.19 d	2113.86 c	2063.92 e	4359.01 e	4017.35 d	0.48 ab	0.47 ab
XY 508	N 240	327.09 cd	452.04 b	2148.36 c	2348.40 d	5147.12 d	4821.77 c	0.48 abc	0.48 a
	N 360	325.66 cd	452.10 b	2290.59 b	2423.10 cd	5601.46 c	4907.12 bc	0.48 ab	0.48 a
	Mean	**303.79 B**	**408.76 B**	**2027.52 B**	**2156.09 B**	**4692.20 B**	**4265.10 B**	**0.48 A**	**0.48 A**
*F* value	Years (Y)	822.43**		70.33**		161.68**		6.35*	
	Cultivar (C)	178.69**		263.66**		534.00**		41.15**	
	Nitrogen (N)	189.88**		287.27**		249.56**		1.70^ns^	
	Y × C	22.42**		3.04^ns^		18.23**		0.84^ns^	
	Y × N	11.28**		8.26**		3.62*		1.56^ns^	
	C × N	17.06**		17.82**		13.27**		2.55^ns^	
	Y × C × N	17.03**		4.38*		3.76*		1.43^ns^	

Values followed by different lowercase letters are significantly different with *p* < 0.05; within cultivars, values with different uppercase letters are significantly different with *p* < 0.05 according to the least significant difference test. **p < 0.01; *p < 0.05; ^ns^Not significant. The bold values are the mean value of one cultivar in different N treatments.

The pre-anthesis C and N accumulation ratios of maize varied between 36.82–53.98% and 54.75–74.93%, respectively, and the post-anthesis C and N accumulation ratios varied between 46.02–63.18% and 25.07–45.25%, respectively (Table 4). Maize had a higher proportion of pre-anthesis N accumulation and a higher proportion of post-anthesis C accumulation. In both years, the proportion of pre-anthesis C and N accumulation was significantly (*p* < 0.01) lower in ZH 311 than in XY 508, whereas the proportion of post-anthesis C and N accumulation and the post-/pre ratio of C and N accumulation were significantly (*p* < 0.01) higher than those of XY 508, indicating that the N-efficient cultivar ZH 311 has stronger post-anthesis C and N accumulation ability than the N-inefficient cultivar XY 508. The N application was beneficial in increasing the proportion of pre-anthesis C and N accumulation while reducing the proportion of post-anthesis C and N accumulation, but there were significant differences between cultivars. Compared with N 0 treatment, the mean N accumulation ratio of N application treatments of ZH 311 was changed by 3.16 and 10.10 percentage points, and the mean C accumulation ratios of N application treatments of ZH 311 were changed by 0.57 and 1.40 percentage points in 2019 and 2020, respectively, while those of XY 508 were changed by 7.44, 10.54, 1.18, and 4.12 percentage points, respectively. ZH 311 had a lower change in proportion of C and N accumulation under N application than XY 508, indicating that the N-inefficient cultivar XY 508 depended more on N application to coordinate the pre- and post-anthesis C and N accumulation than the N-efficient cultivar ZH 311.

**TABLE 4:** Differences of C and N accumulation proportions pre- and post-anthesis and post-/pre- ratio under different treatments.

Cultivar	Nitrogen rate	Nitrogen accumulation			Carbon accumulation		
		Pre-anthesis (%)	Post-anthesis (%)	Post-/Pre-	Pre-anthesis (%)	Post-anthesis (%)	Post-/Pre-
**2019**							
	N 0	63.58 b	36.42 a	0.57 a	36.82 e	63.18 a	1.72 a
	N 120	62.18 b	37.82 a	0.61 a	38.03 e	61.97 a	1.63 b
ZH 311	N 240	72.11 a	27.89 b	0.39 d	39.63 d	60.37 b	1.52 c
	N 360	65.94 b	34.09 a	0.52 c	37.03 e	62.97 a	1.70 ab
	Mean	**65.95 B**	**34.05 A**	**0.52 A**	**37.88 B**	**62.12 A**	**1.64 A**
	N 0	65.76 b	34.24 a	0.52 c	42.52 b	57.48 d	1.35 e
	N 120	74.93 a	25.07 b	0.33 e	48.48 a	51.52 e	1.06 f
XY 508	N 240	72.86 a	27.14 b	0.37 d	41.74 bc	58.26 cd	1.39 de
	N 360	71.80 a	28.20 b	0.39 d	40.89 cd	59.11 bc	1.45 d
	Mean	**71.34 A**	**28.66 B**	**0.40 B**	**43.41 A**	**56.59 B**	**1.31 B**
**2020**							
	N 0	54.75 d	45.25 a	0.83 a	48.45 c	51.55 a	1.06 ab
	N 120	64.05 bc	35.95 bc	0.56 bc	48.20 c	51.80 a	1.07 ab
ZH 311	N 240	65.34 b	34.66 c	0.53 cd	47.82 c	52.18 a	1.09 ab
	N 360	65.17 b	34.83 c	0.53 cd	47.64 c	52.36 a	1.10 a
	Mean	**62.33 B**	**37.67 A**	**0.61 A**	**48.03 B**	**51.97 A**	**1.08 A**
	N 0	55.82 d	44.18 a	0.79 a	53.98 a	46.02 c	0.85 e
	N 120	68.85 a	31.15 d	0.45 e	51.46 b	48.54 b	0.95 d
XY 508	N 240	68.14 a	31.86 d	0.47 de	48.74 c	51.26 a	1.05 bc
	N 360	62.10 c	37.90 b	0.61 b	49.37 c	50.63 a	1.02 c
	Mean	**63.73 A**	**36.27 B**	**0.58 B**	**50.89 A**	**49.11 B**	**0.97 B**
*F* value	Years (Y)	54.93**	54.93**	216.99**	983.63**	983.63**	2266.62**
	Cultivar (C)	20.06**	20.06**	65.16**	223.14**	223.14**	535.78**
	Nitrogen (N)	29.93**	29.93**	126.01**	18.70**	18.70**	35.99**
	Y × C	6.95*	6.95*	22.07**	22.54**	22.54**	131.92**
	Y × N	3.88*	3.88*	26.56**	15.30**	15.30**	33.45**
	C × N	5.61**	5.61**	18.14**	19.19**	19.19**	39.98**
	Y × C × N	3.09*	3.09*	10.43**	7.70**	7.70**	16.10**

Values followed by different lowercase letters are significantly different with *p* < 0.05; within cultivars, values with different uppercase letters are significantly different with *p* < 0.05 according to the least significant difference test. **p < 0.01; *p < 0.05. The bold values are the mean value of one cultivar in different N treatments.

### Differences in balance of carbon and nitrogen of maize

The C/N ratio of maize increased gradually with growth delay (Table 5). It was 11.45–21.24 at the jointing stage, 20.99–42.63 at the silking stage, and 55.61–90.88 at the maturity stage. The C/N ratio of XY 508 was significantly (*p* < 0.01) higher than that of ZH 311 at each stage. In 2019 and 2020, it was higher by 5.65 and 1.54% at the jointing stage, 10.56 and 6.29% at the silking stage, 6.36 and 6.00% at the maturity stage, and the mean value of each stage was higher by 7.30 and 5.52%, respectively. The difference in C/N ratio between ZH 311 and XY 508 was found to be the highest in the silking stage. The C/N ratio of maize decreased significantly (*p* < 0.01) with N application, but there were clear differences in reduction between cultivars. Compared with N 0 treatment, in 2019 and 2020, the mean C/N ratio of N application treatments of ZH 311 decreased by 41.05 and 32.37% in the jointing stage, 26.96 and 29.80% in the silking stage, and 23.28 and 25.64% in the maturity stage, respectively; those of XY 508 decreased by 41.81 and 34.99%, 33.23 and 37.62%, and 25.93 and 26.31%, respectively. The reduction of the C/N ratio in each stage of XY 508 with N application was higher than that of ZH 311, indicating that the N-inefficient cultivar XY 508 is more reliant on N application than the N-efficient cultivar ZH 311 to coordinate the C/N ratio of the plants, especially in the silking stage.

**TABLE 5:** Differences of C/N ratios of whole maize plant at different stages.

Cultivars	Nitrogen rate	Jointing stage		Silking stage		Maturity stage		Mean	
		2019	2020	2019	2020	2019	2020	2019	2020
	N 0	19.95 b	18.06 b	32.54 b	37.08 b	79.27 b	85.99 b	43.92 b	47.04 b
	N 120	12.35 cd	12.91 c	29.12 d	30.40 c	66.42 d	74.86 d	35.96 d	39.39 d
ZH 311	N 240	11.47 e	11.85 e	20.99 f	24.14 e	59.36 f	61.36 f	30.61 g	32.45 f
	N 360	11.45 e	11.88 e	21.19 f	23.55 e	56.66 g	55.61 g	29.77 g	30.34 g
	Mean	**13.80 B**	**13.67 B**	**25.96 B**	**28.79 B**	**65.43 B**	**69.46 B**	**35.06 B**	**37.31 B**
	N 0	21.24 a	18.82 a	38.23 a	42.63 a	86.40 a	90.88 a	48.62 a	50.78 a
	N 120	12.93 c	12.78 c	30.86 c	30.19 c	68.84 c	81.95 c	37.48 c	41.64 c
XY 508	N 240	12.46 cd	11.63 e	23.19 e	25.63 d	62.40 e	64.79 e	32.69 e	34.02 e
	N 360	11.68 de	12.29 d	22.52 e	23.95 e	60.93 e	56.89 g	31.71 f	31.04 g
	Mean	**14.58 A**	**13.88 A**	**28.70 A**	**30.60 A**	**69.59 A**	**73.63 A**	**37.62 A**	**39.37 A**
*F* value	Years (Y)	14.03**		170.23**		174.44**		171.09**	
	Cultivar (C)	19.56**		157.63**		186.62**		229.37**	
	Nitrogen (N)	1122.20**		1455.49**		1690.94**		2499.91**	
	Y × C	6.50*		6.64*		0.00^ns^		2.66^ns^	
	Y × N	29.27**		22.98**		85.92**		28.55**	
	C × N	2.68^ns^		39.68**		5.75**		18.00**	
	Y × C × N	1.71^ns^		1.08^ns^		8.43**		2.04^ns^	

Values followed by different lowercase letters are significantly different with *p* < 0.05; within cultivars, values with different uppercase letters are significantly different with *p* < 0.05 according to the least significant difference test. **p < 0.01; *p < 0.05; ^ns^Not significant. The bold values are the mean value of one cultivar in different N treatments.

The stem + sheath and leaf C/N ratios of maize increased with growth, and were highest at maturity stage in both years, while the root C/N ratio was highest at silking stage in 2019 and at maturity stage in 2020 (Table 6). In both years, the mean value of all stages in the root, stem + sheath, and leaf C/N ratio of ZH 311 was lower than that of XY 508. The difference in root and leaf C/N ratios between two cultivars reached a significant level (*p* < 0.01) in 2019, which was lower by 4.52 and 6.84%, respectively. The difference of stem + sheath and leaf C/N ratios between two cultivars reached a significant level (*p* < 0.01) in 2020, which was lower by 4.04 and 7.08%, respectively, whereas the difference between two cultivars of stem + sheath C/N ratio in 2019 and that of root C/N ratio in 2020 was not significant, indicating that the C/N ratio in each vegetative organ of ZH 311 was lower than that of XY 508, and the difference between the two cultivars in root and stem + sheath C/N ratio was affected by year, so the difference between the two cultivars in leaf C/N ratio was the highest in vegetative organs. The C/N ratio decreased significantly with N application in various organs and stages of maize, and there was a significant interaction effect between cultivar and N application. Compared with N 0 treatment, the mean value of N application treatments in root, stem + sheath, and leaf C/N ratio of ZH 311 decreased by 36.54, 35.16, and 19.80% in 2019, and decreased by 29.99, 34.41, and 24.39% in 2020, respectively, and those of XY 508 decreased by 40.62, 41.55, and 28.04% in 2019, and 42.79, 37.13, and 29.22% in 2020, respectively. Increases in C/N ratio in XY 508 vegetative organs with N application were significantly higher than those of ZH 311, indicating that the N-inefficient cultivar XY 508 was more dependent on N application to coordinate vegetative organ C/N ratio than the N-efficient cultivar.

**TABLE 6:** Differences of C/N ratios in vegetative organs of maize at different stages.

Cultivar	Nitrogen rate	Root				Stem + sheath				Leaf			
		Jointing stage	Silking Stage	Maturity stage	Mean	Jointing stage	Silking Stage	Maturity stage	Mean	Jointing stage	Silking Stage	Maturity stage	Mean
2019													
	N 0	29.87 b	50.82 b	41.67 a	40.79 b	16.68 a	52.98 b	81.09 b	50.25 b	13.29 a	15.08 a	29.25 b	19.20 b
	N 120	18.63 d	42.65 c	29.93 c	30.40 c	8.47 c	49.61 c	66.38 d	41.48 d	9.94 b	12.50 bc	27.99 c	16.81
ZH 311	N 240	16.21 e	28.02 e	23.92 d	22.72 e	8.03 c	30.86 e	62.31 e	33.73 f	10.16 b	10.68 d	22.99 e	14.61 f
	N 360	15.96 e	28.17 e	21.95 d	22.03 e	8.20 c	34.34 d	57.54 f	33.36 f	10.20 b	10.92 d	23.24 e	14.78 f
	Mean	**20.17 B**	**37.41 B**	**29.37 A**	**28.98 B**	**10.35 A**	**41.95 B**	**66.83 B**	**39.71 B**	**10.90 A**	**12.29 B**	**25.87 B**	**16.35 B**
	N 0	35.40 a	61.05 a	37.34 b	44.60 a	14.58 b	65.14 a	90.73 a	56.82 a	13.72 a	15.22 a	37.72 a	22.22 a
	N 120	21.14 c	42.18 c	27.83 c	30.38 c	8.76 c	50.20 c	74.51 c	44.49 c	8.90 c	13.04 b	29.07 b	17.00 c
XY 508	N 240	20.52 cd	31.48 d	24.33 d	25.44 d	8.14 c	33.33 de	67.70 d	36.39 e	8.73 c	12.32 bc	26.08 d	15.71 d
	N 360	19.72 cd	30.79 de	23.08 d	24.53 d	7.27 d	33.71 d	65.39 d	35.46 e	8.06 c	11.93 c	25.79 d	15.26 e
	Mean	**24.20 A**	**41.38 A**	**28.14 A**	**31.24 A**	**9.68 B**	**45.60 A**	**74.58 A**	**43.29 A**	**9.85 B**	**13.13 A**	**29.67 A**	**17.55 A**
2020													
	N 0	23.27 b	34.03 b	47.09 a	34.80 b	16.20 a	69.68 b	91.65 b	59.18 b	14.70 a	22.84 b	43.30 b	26.95 b
	N 120	15.97 c	33.98 b	32.39 b	27.44 c	10.86 c	53.83 c	84.40 c	49.70 d	11.88 v	14.96 d	37.88 c	21.58 d
ZH 311	N 240	14.67 d	27.05 ef	26.33 c	22.69 ef	9.79 ef	38.64 d	63.45 d	37.29 e	11.09 de	12.62 f	31.26 e	18.33 fg
	N 360	15.73 c	26.57 f	22.55 d	21.62 f	9.75 f	34.77 e	46.97 f	30.50 g	10.15 f	12.81 f	33.44 d	18.80 g
	Mean	**17.41 B**	**30.41 B**	**32.09 B**	**26.64 B**	**11.65 A**	**49.23 B**	**71.62 B**	**44.17 B**	**11.96 A**	**15.81 B**	**36.47 B**	**21.41 B**
	N 0	29.33 a	38.63 a	48.41 a	38.79 a	13.51 b	82.79 a	97.75 a	64.74 a	13.61 b	24.26 a	47.35 a	28.41 a
	N 120	15.84 c	28.98 d	34.34 b	26.39 c	11.15 c	55.46 c	92.56 b	53.06 c	11.34 d	15.78 c	43.54 b	23.55 c
XY 508	N 240	13.63 e	31.00 c	27.41 c	24.01 d	10.68 cd	38.46 d	60.82 d	36.65 e	10.60 ef	13.71 e	38.32 c	20.88 e
	N 360	15.79 c	28.53 de	24.99 cd	23.10 de	10.31 de	37.12 de	51.53 e	32.99 f	10.78 e	12.79 f	34.34 d	19.30 f
	Mean	**18.64 A**	**31.78 A**	**33.79 A**	**28.07 A**	**11.41 A**	**53.50 A**	**75.66 A**	**46.86 A**	**11.58 B**	**16.64 A**	**40.89 A**	**23.04 A**
*F* value	Years (Y)	247.65**	438.92**	87.42**	128.23**	178.45**	241.42**	20.99**	161.18**	115.33**	801.69**	2711.59**	3759.48**
	Cultivar (C)	99.22**	45.44**	0.28^ns^	57.46**	15.78**	65.71**	84.83**	98.39**	29.91**	45.08**	383.86**	268.58**
	Nitrogen (N)	565.39**	427.27**	418.22**	1019.53**	701.75**	1026.62**	599.73**	1269.64**	205.82**	739.73**	524.01**	1470.09**
	Y × C	27.92**	10.64**	10.68**	2.82^ns^	3.37^ns^	0.40^ns^	8.39**	1.98^ns^	6.59*	0.00^ns^	2.14^ns^	6.08*
	Y × N	10.85**	123.71**	11.29**	25.12**	45.42**	47.11**	106.66**	73.00**	5.94**	177.66**	9.97**	59.27**
	C × N	16.28**	28.15**	2.41^ns^	14.00**	34.17**	35.78**	5.98**	11.51**	1.10^ns^	2.30^ns^	22.45**	20.43**
	Y × C × N	5.48**	3.48*	1.71^ns^	0.50^ns^	4.04*	1.46^ns^	1.66^ns^	1.87^ns^	11.51**	4.16*	27.31**	19.65**

Values followed by different lowercase letters are significantly different with *p* < 0.05; within cultivars, values with different uppercase letters are significantly different with *p* < 0.05 according to the least significant difference test. **p < 0.01; *p < 0.05; ^ns^Not significant. The bold values are the mean value of one cultivar in different N treatments.

### Differences in maize harvest index, yield, and yield components

The effects of cultivar, N level, and year on maize grain yield, yield composition, and harvest index were significant (*p* < 0.01, Table 7). The kernels per ear and grain yield of ZH 311 were significantly higher than those of XY 508 in both years, with the kernels per ear being higher by 24.73 and 19.92% in 2019 and 2020, respectively, and the grain yield being higher by 12.51 and 13.45%, respectively, while the 1,000-kernel weight and harvest index of ZH 311 were significantly lower than those of XY 508 in both years, with the 1,000-kernel weight being lower by 5.51 and 4.26%, and the harvest index being lower by 2.35 and 3.18%, respectively. The kernels per ear, 1,000-kernel weight, and grain yield of maize increased significantly with N application, while the harvest index decreased significantly. There was a significant interaction effect between N application and cultivar on kernels per ear, 1,000-grain weight, and grain yield of maize (*p* < 0.01). The kernels per ear, 1,000-grain weight, and grain yield of ZH 311 increased first and then decreased with increasing N level, with N 240 being the highest in both years, while the kernels per ear, 1,000-grain weight, and grain yield of XY 508 increased with increasing N application, with N 360 being the highest in both years. The regression equations of average grain yield in both years (*y*) and N level (*x*) for ZH 311 and XY 508 were *y*_*ZH311*_ = –0.000028 *x*^2^+0.0153 *x+*5.6455 (*R*^2^ = 0.9315^**^) and *y*_*XY508*_ = –0.000017 *x*^2^+0.0121 *x+*4.8615 (*R*^2^ = 0.9984^**^). The lowest and highest grain yield of N-efficient cultivar ZH 311 (5.65 t ha^–1^ and 7.74 t ha^–1^) were significantly higher than those of N-inefficient cultivar (4.86 t ha^–1^ and 7.01 t ha^–1^), while the optimum N level when obtaining the highest grain yield of ZH 311 (273.21 kg ha^–1^) was significantly lower than that of XY 508 (355.88 kg ha^–1^).

**TABLE 7:** Effects of nitrogen application on harvest index, yield, and yield components of maize.

Cultivars	Nitrogen rate	Kernels per ear		1,000-kernel weight (g)		Grain yield (t ha^–1^)		Harvest index (%)	
		2019	2020	2019	2020	2019	2020	2019	2020
ZH 311	N 0	366.66 d	337.96 d	271.87 g	266.75 g	5.94 e	5.48 e	0.44 cd	0.46 c
	N 120	437.96 b	411.18 b	283.38 e	280.07 e	7.24 d	6.55 bc	0.44 bcd	0.45 cd
	N 240	468.39 a	448.11 a	291.68 d	290.78 c	8.24 a	7.46 a	0.43 d	0.44 d
	N 360	458.34 a	431.63 a	285.04 e	284.56 d	7.97 b	6.75 b	0.43 d	0.42 e
	Mean	**432.84 A**	**407.22 A**	**282.99 B**	**280.54 B**	**7.35 A**	**6.56 A**	**0.44 B**	**0.44 B**
XY 508	N 0	283.47 f	281.07 e	279.55 f	277.23 f	5.20 f	4.64 f	0.47 a	0.49 a
	N 120	325.17 e	338.60 d	300.37 c	291.76 c	6.10 e	5.71 e	0.46 ab	0.48 ab
	N 240	379.06 d	361.58 c	310.25 b	299.45 b	7.11 d	6.25 d	0.46 ab	0.46 bc
	N 360	400.41 c	377.02 c	316.61 a	303.58 a	7.46 c	6.47 cd	0.46 abc	0.45 cd
	Mean	**347.03 B**	**339.57 B**	**301.70 A**	**293.01 A**	**6.47 B**	**5.77 B**	**0.46 A**	**0.47 A**
*F* value	Years (Y)	36.60**		156.60**		437.75**		2.69^ns^	
	Cultivar (C)	787.57**		1224.54**		559.01**		71.81**	
	Nitrogen (N)	287.96**		640.70**		642.14**		7.02**	
	Y × C	11.03**		49.00**		1.60^ns^		0.07^ns^	
	Y × N	1.96^ns^		2.12^ns^		15.06**		5.83**	
	C × N	9.40**		59.60**		21.60**		0.62^ns^	
	Y × C × N	2.81^ns^		14.27**		2.00^ns^		0.12^ns^	

Values followed by different lowercase letters are significantly different with *p* < 0.05; within cultivars, values with different uppercase letters are significantly different with *p* < 0.05 according to the least significant difference test. **p < 0.01; *p < 0.05; ^ns^Not significant. The bold values are the mean value of one cultivar in different N treatments.

## Discussion

### Correlation between nitrogen accumulation and grain yield in maize

**FIGURE 2:**
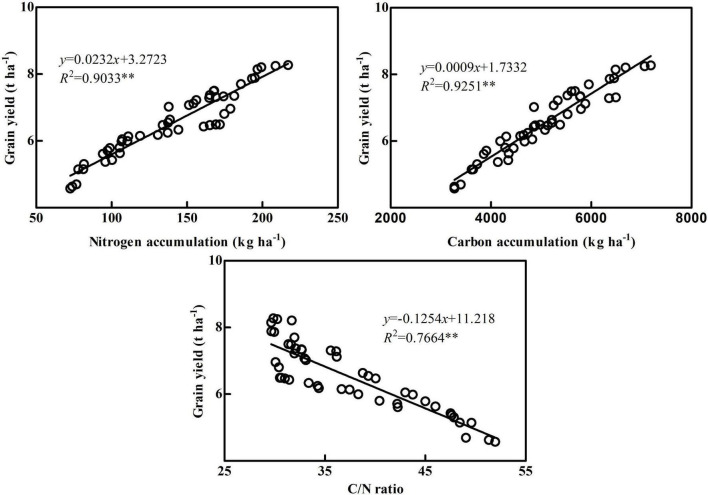
Correlation analysis between grain yield and carbon accumulation, nitrogen accumulation, and C/N ratio. The data used for correlation analysis were nitrogen accumulation at maturity, carbon accumulation at maturity, and mean value of C/N at JSB, SS, and MS. **Significant level of correlation.

**FIGURE 3:**
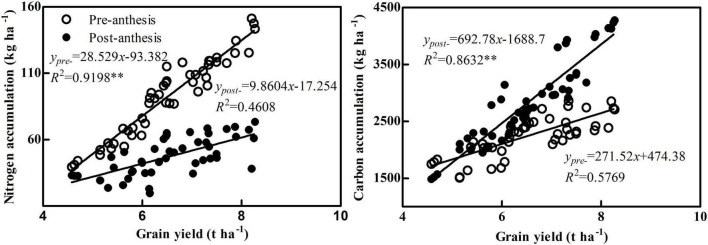
Correlation analysis between grain yield and pre- and post-anthesis accumulation of nitrogen and carbon. **Significant level of correlation.

Grain N accumulation in maize maturity caused by two factors: the transport of pre-anthesis and post-anthesis N accumulation, so plant N accumulation directly affects the maize grain yield (Wang et al., 2020b; Liu et al., 2021). Under N deficiency, maize will increase the transport of pre-anthesis N accumulation to ensure grain yield, and increasing N application can improve post-anthesis N accumulation and its contribution to grain, resulting in a high yield (Wu et al., 2021). Chun et al. (2005) pointed out that the N accumulation of N-efficient cultivars (NE 1 and ND 108) was significantly higher than that of N-inefficient cultivars, resulting in significantly higher grain yield of N-efficient cultivars. The results showed that N accumulation was significantly higher in each stage of the N-efficient cultivar ZH 311 than in the N-inefficient cultivar XY 508, which was consistent with previous research results (Wang et al., 2020a; Liu et al., 2021), N-efficient cultivars take up more N than N-inefficient cultivars. Moreover, the N accumulation of ZH 311 increased first and then decreased with the increase of N application, whereas the N accumulation of XY 508 increased with the increase of N application, indicating that excessive N application of N-efficient cultivar ZH 311 will affect the N accumulation, and high N application is conductive to increasing the N accumulation of N-inefficient cultivar XY 508, which was contradictory to previous results on N utilization efficient maize cultivars (Zhang, 2013; Chang et al., 2017). ZH 311 is a N absorption efficient cultivar with increased root N absorption ability, and its N accumulation and grain yield advantages are greater at low and medium N levels (Li et al., 2017, 2019). Therefore, the difference in N accumulation and grain yield between ZH 311 and XY 508 increases first and then decreased with the increase of N application, which is consistent with the findings reported by Wang et al. (2020a) using low N-efficient cultivar Jingnongke 728. Correlation analysis showed that grain yield was positively correlated with N accumulation (*R*^2^ = 0.9033^**^, Figure 2), indicating that high N accumulation was the source of the high grain yield of maize. Further analysis showed that grain yield was significantly positively correlated with pre-anthesis N accumulation (*R*^2^ = 0.9198^**^) but not with post-anthesis N accumulation (*R*^2^ = 0.4608, Figure 3). Pre-anthesis accounts for 54.75–74.93% of the N accumulation of a maize plant, and higher pre-anthesis N accumulation helps in the establishment of plant vegetative organs and promotes post-anthesis photosynthetic product accumulation, resulting in a high yield, which is consistent with the previous results (Jin and He, 1999; Chang et al., 2017; Wu et al., 2021).

### Correlation between carbon accumulation and grain yield in maize

Carbohydrates account for more than 90% of maize dry matter, making C accumulation in plants a basis for maize grain yield formation (Liang et al., 2019). Ding (2005) compared the photosynthetic characteristics of maize cultivars at different ages and found that the photosynthetic rate and high photosynthetic period of new cultivars were improved compared with old cultivars, resulting in significantly higher C accumulation and grain yield of new cultivars. The results showed that C accumulation in each stage of the N-efficient cultivar ZH 311 was significantly higher than in the N-inefficient cultivar XY 508, which was consistent with the results of Wu et al. (2021). Furthermore, the C accumulation of ZH 311 increased first and then decreased with increasing N level, while that of XY 508 increased with increasing N level, indicating that excessive N metabolism of N-efficient cultivar ZH 311 will affect the C accumulation, and high N application is conductive to improving the C accumulation of N-inefficient cultivar XY 508. In a certain range, N application level was positively correlated with C accumulation, while excessive N application reduced the C assimilation rate of maize (Zhang et al., 2015; Zhang, 2019). Excess N application increases the N assimilation intensity in photosynthetic organs, which competes with C assimilation for ATP and NADPH, reducing C accumulation (Cheng et al., 2021). Correlation analysis revealed that maize grain yield was significantly positively correlated with C accumulation (*R*^2^ = 0.9251^**^, Figure 2), indicating that maize C accumulation ability affects maize grain yield, which was consistent with the findings of Tian (2013). Further analysis found that grain yield was not significantly correlated with pre-anthesis C accumulation (*R*^2^ = 0.5769) but was significantly related to post-anthesis C accumulation (*R*^2^ = 0.8632^**^, Figure 3). Post-anthesis accounts for 54.75–74.93% of C accumulation of maize plants, and it accounts for most of the C accumulation in grains (74.8–92.8%). Therefore, post-anthesis C accumulation has a greater impact on grain yield than pre-anthesis C accumulation, which is in line with findings from previous studies (Jin and He, 1999; Dai et al., 2011).

### Correlation between balance of carbon and nitrogen and grain yield in maize

**FIGURE 4:**
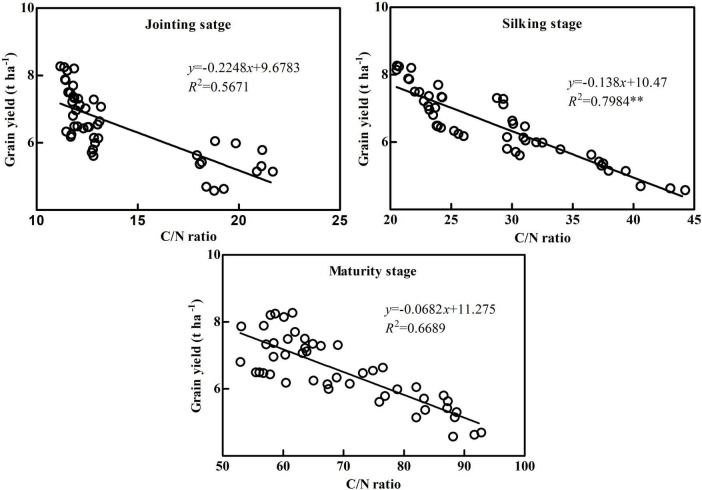
Correlation analysis between grain yield and C/N ratios at different stages. **Significant level of correlation.

**FIGURE 5:**
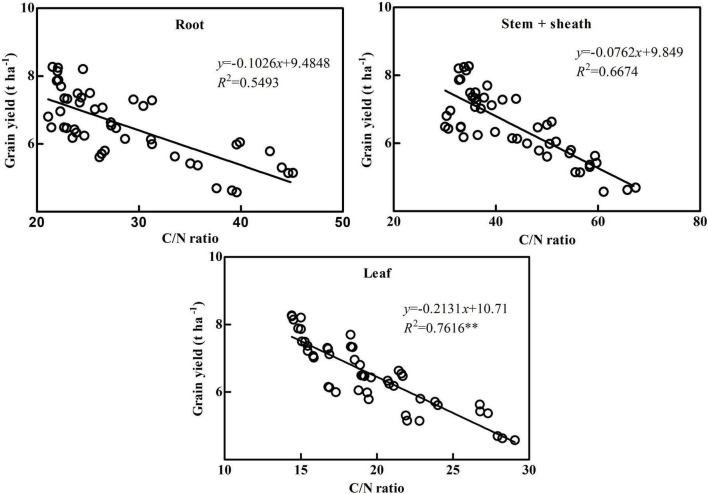
Correlation analysis between grain yield and C/N ratio of different organ. **Significant level of correlation.

C and N metabolisms interact and inhibit each other. N metabolism requires C metabolism for C source and energy, while C metabolism needs N metabolism for enzymes and photosynthetic pigments (Commichau et al., 2006). At the early growth stage, plant root absorption, photosynthesis and other accumulation metabolism are strong, which makes a large of nutrients such as C and N accumulation in vegetative organs, and the ratio of carbon to nitrogen is low. In the reproductive period, photosynthetic assimilation capacity of leaves decreased, root absorption capacity decreased, soil nutrients decreased, energy and material competition between organs increased, and C/N ratio increased. At mature stage, nutrient transfer and C/N ratio increase. Lower C/N ratio can delay vegetative organs senescence and improve photosynthetic efficiency (Yu et al., 2014; Zuo et al., 2016). Therefore, only a proper balance of C and N metabolism can achieve the purpose of increasing grain yield (Liang et al., 2019). The C/N ratio can reflect the balance of C and N metabolism in plants, but there are significant differences between organs and growth stages (Dai et al., 2011). The results showed that the C/N ratio of maize plants increased significantly with the delay of the growth process, indicating that maize pre-anthesis was dominated by N metabolism, and post-anthesis was dominated by C metabolism, which was consistent with the previous results (Zhang et al., 2016; Zuo et al., 2016). In terms of organs, the C/N ratio in each stage of leaf was the lowest among vegetative organs, indicating that leaf is the main vegetative organ for coordinating C and N balance in maize. The C/N ratio of the leaf was too low and not conducive to photosynthetic product storage and transport, while it was too high and not conducive to maintaining leaf function (Chang et al., 2017). The results showed that the C/N ratio of ZH 311 was significantly lower than that of XY 508 at different stages and vegetative organs, with the greatest difference in C/N ratio occurring at the silking stage and in the leaf. The C/N ratio in ZH 311 leaves was significantly higher than that of XY 508 at the jointing stage in both years, while the C/N ratio in ZH 311 leaves was significantly lower than that of XY 508 at the silking stage and maturity stage in both years. The higher pre-anthesis C/N ratio in the leaf was beneficial to storage and transport of photosynthetic products, while the lower post-anthesis C/N ratio in the leaf was conducive to prolonging the leaf functional period, which is an important reason why the C and N accumulation of the N-efficient cultivar ZH 311 was significantly higher than that of the N-inefficient cultivar XY 508. Correlation analysis revealed that grain yield was significantly negatively correlated with the C/N ratio (*R*^2^ = 0.7664^**^, Figure 2), indicating that the balance of C and N could regulate maize grain yield, which was consistent with the results of Dai et al. (2011). Further analysis found that grain yield was negatively correlated with the C/N ratio in different stages and vegetative organs. The correlation between grain yield and C/N ratio in the silking stage was the highest (*R*^2^ = 0.7984^**^, Figure 4) among different stages, and that in the leaf was the highest (*R*^2^ = 0.7616^**^, Figure 5), indicating that the balance of C and N in different stages and organs had an impact on maize grain yield, but the balance of C and N in the silking stage and leaf had the highest impact on maize grain yield. We have clarified the correlation between the accumulation and balance of C and N and grain yield in maize, but the impact of cultivation measures on C and N accumulation and balance during the production process requires further research.

## Conclusion

Identifying the correlation between the accumulation and balance of C and N and grain yield provides a theoretical basis and technical support for the breeding and cultivation management of N-efficient maize cultivars. ZH 311 had significantly higher C and N accumulation and grain yield than XY 508. The C and N accumulation and grain yield of both cultivars significantly increased with N application, while the differences in C and N accumulation and grain yield between ZH 311 and XY 508 increased first and then decreased with N level increase, indicating that the N-efficient cultivar ZH 311 had greater advantages in C and N accumulation and grain yield under low and medium N levels, while a high N level favored the N-inefficient cultivar XY 508. The C/N ratio of ZH 311 was significantly lower than that of XY 508, and the difference in the C/N ratio between both cultivars was highest in the silking stage and leaf, indicating that the N-efficient cultivar ZH 311 can better coordinate the C and N balance of the plant than the N-inefficient cultivar XY 508, especially in the silking stage and leaf. Furthermore, correlation analysis revealed that grain yield was significantly positively correlated with C and N accumulation and was mainly affected by pre-anthesis N accumulation and post-anthesis C accumulation; while it was significantly negatively correlated with the C/N ratio, and the correlation between grain yield and the C/N ratio in silking stage and leaf was the highest. In comparison to the N-inefficient cultivar XY 508, the N-efficient cultivar ZH 311 can better coordinate the C and N balance of plants, particularly the C and N balance in the silking stage and leaf, promote photosynthetic product storage and transport, and prolong the leaf function period, resulting in significantly higher pre-anthesis and post-anthesis C and N accumulation and a high grain yield.

## Data availability statement

The raw data supporting the conclusions of this article will be made available by the authors, without undue reservation.

## Author contributions

QL and JY designed the study. YR, ZL, and HF performed the experiments. YR and HF analyzed the data. FK and HF developed the new methods. QL wrote the manuscript. All authors listed have made a substantial, direct, and intellectual contribution to the work, and approved it for publication.

## Abbreviations

ZH 311: Zhenghong 311
XY 508: Xianyu 508
JS: jointing stage
SS: silking stage
MS: maturity stage.
